# Green Growth, Green Technology, and Environmental Health: Evidence From High-GDP Countries

**DOI:** 10.3389/fpubh.2021.816697

**Published:** 2022-01-14

**Authors:** Zahid Hussain, Bilal Mehmood, Muhammad Kaleem Khan, Raymondo Sandra Marcelline Tsimisaraka

**Affiliations:** ^1^School of Finance, Qilu University of Technology (Shandong Academy of Sciences), Jinan, China; ^2^School of Economics, Director Centre for Economics and Business Research, University of the Punjab, Lahore, Pakistan; ^3^Asia-Australia Business College, Liaoning University, Shenyang, China; ^4^Business School, Huanggang Normal University, Huanggang, China

**Keywords:** green growth, green technology, energy consumption, environment, GDP

## Abstract

Green growth is an exceptional strategy for sustainable development. It provides a pathway to combat environmental issues and the use of natural resources. This study investigates the effects of green technology and environmental factors on green growth in high-gross domestic product (GDP) countries from 2000 to 2020. In addition, it also probes the linear and nonlinear effects of GDP on green growth. To do so, we employ an advanced econometric approach, e.g., a cross-sectional autoregressive distributed lags estimator for long and short runs. The outcomes demonstrate that the linear effect of GDP is positive for green growth. On the contrary, the nonlinear effect of GDP has a negative magnitude for green growth. Besides, green technology substantially increases green growth. Energy consumption is found to be an important influencer, and it decreases green growth. Environmental factors such as emissions, according to the findings, also reduce green growth in the sample countries. It is worth noting that the joint effects of energy consumption and emissions deteriorate green growth in countries. Based on empirical findings, for policy makers, this study suggests that high-GDP countries should manage their economic and environmental activities in order to increase the amount of green growth that may protect the ecological environment.

## Introduction

The term “green growth” has been used to refer to ecological protection. An American marine biologist (R. Carson) published an article entitled “Silent Spring” in 1987, demonstrating that excessive pesticide input would have a detrimental effect on the environment and people's understanding of the harmful effects caused by pollution on human development. It has had significant global ramifications. Besides, a British economist, namely Pierce, introduced the notion of “green economy” for the first time in 1989 with the publication of the “Green Economy Blue Book.” The author explains that natural resource depletion will cause economic growth to permanently stagnate if economic expansion exceeds the limit of available natural resources (1, 2).

Green growth has received a lot of attention since the consideration of climate change and environmental degradation. Several organizations, including the World Bank, the Organization for Economic Cooperation and Development (OECD), and the United Nations Economic and Social Commission for Asia and the Pacific (UNESCAP), are taking the green economy seriously.The reason for the continuous rise in global temperature and its concomitant effect on the planet is that it has been prioritized in the intergovernmental panel on climate change (3, 4). In order to address the climate change issues, the Paris Agreement and the 2030 Sustainable Development Agenda have renewed the actions toward a better environment (5). Indeed, the (5) suggests that Agenda 2030 for Sustainable Development is the norm for all countries, developed or developing. There are substantial discrepancies between which countries have a concentration on environmentally sustainable economic development. However, high-gross domestic product (GDP) countries are committed to an eco-friendly environment. The high-GDP countries are experiencing a significant increase in green growth, but lower-income countries are stagnant or declining in green growth. Therefore, with a rise in continuous economic activity, it indicates that there are multifaceted environmental issues that cannot be solved by all the strategies.

We assess the environmental factors and green technology that affect green growth. According to the (5), green growth indicates whether economic growth is becoming greener with more efficient use of natural capital. The green growth indicator [environmentally adjusted multifactor productivity (EAMFP)] measures advancements concerning the sustainable and greener economy (5). Several researchers have started investigations into green growth and its influencers. For instance, (6) argue that advancements in green technology could play a supportive role in green growth. Cao and Bai (7) analyze that there is a relationship between green growth and the environment. Subsequently, (6, 8, 9) concluded that technological innovation, technological progress related to the environment, and green industrial development have a remarkable impact on green growth (10). Besides, (11) studied the environmental factors that substantially affect green growth.

This article addresses the challenges regarding green growth that still exist and vary from country to country due to the diverse economic system and environmental policies. Thus, countries need to identify the factors that influence green growth and provide better strategies to tackle the issues. More analytically, environmental taxes (ENTs) have a substantial impact on green growth. In explaining, such factors encourage the market's concern over economic activities (12). Similarly, emissions are also imperative in playing a productive role in green growth. Some high-GDP countries have the potential to spend a larger amount of national income on the environment in order to tackle the undesired outputs and preserve the natural base assets, whereas poor countries are lagging behind in tackling the environmental issues due to the lack of funds. Besides, energy consumption also deteriorates green growth through the use of fossil fuels (oil, coal, and gas), which are discovered from natural resources. Ultimately, the inverse impact of environmental factors on energy consumption and the rapidly increasing burden of ENTs appear.

This study investigates whether environmental factors and green technology affect green growth in high-GDP countries. In addition, we question how environmental factors, e.g., emissions, influence green growth through the use of energy consumption. Furthermore, does green technology influence green growth? What strategies should be adopted to avoid the reduction of green growth in order to make it environmentally friendly?

Why high-GDP economies? For some reasons, the research concentrates on high-GDP economies. For instance, economies significantly contribute to the world's GDP. These economies are highly responsible for deteriorating green growth due to continuous economic growth. Economies have an abundance of financial resources to invest in mega projects. Their economic complexity index is high compared with low-GDP economies. Because of a lack of evidence in the current literature, the exclusion of significant factors requires further investigation to explicitly understand the association between green growth and environmental factors. Last, whether environmental factors affect the relationship between energy consumption and green growth is still unclear.

This article motivates by making several contributions to the existing literature: first, examining the role of green technology in green growth, e.g., patents. Second, we included the environmental factors in order to investigate the impact on green growth, i.e., (i) environmental productivity, e.g., carbon dioxide (CO_2_), sulfur dioxde (SO_2_), nitrogen dioxide (NO2), and phosphorus (PH_15_). Previous studies did not emphasize environmental productivity, particularly sulfur dioxide, nitrogen, and phosphorus, but we explored simultaneously the exact identification of challenges for the concerned countries and (ii) ENTs. Third, this study also estimates the linear and nonlinear effects of GDP on green growth. Furthermore, the joint effect of energy consumption and emissions is also investigated in the current model. Last, this research employs an advanced econometric approach, e.g., Cross-Sectionally Augmented Autoregressive Distributed Lag (CS-ARDL), for long-run and short-run estimation.

The findings are documented: green technology enhances green growth by approximately 13.8% in the long run. Likewise, primarily, GDP has substantially impacted green growth. In contrast, the nonlinear effect of GDP reduces green growth. Furthermore, environmental factors, such as emissions, harmed green growth in high-GDP countries. Based on empirical findings, countries should focus on economic activities rather than environmental concerns. Finally, high-GDP countries can tackle the issues related to green growth by decoupling economic growth from GDP growth.

The structure of this article is described as follows: after the introduction, we explained the concept, model estimation, and econometric approaches, including data sources, in the next section, “methodology.” Subsequently, empirical results and interpretations are described in the section titled “Empirical Results.” Section 4 provides discussions of the findings. Finally, we concluded the discussions by identifying limitations and making recommendations for policy implications and future research.

## Review of Prior Studies

The impact of green growth on sustainable development, employment, climate change, and environmental quality has received a lot of attention. However, there is contradictory evidence to back up these assertions (1, 2, 13–16). Also, there are enormous factors that influence green growth, which are considered in the literature (17–19). GDP, ENTs, energy consumption, emissions, and green patents are all imperative determinants of green growth. However, there is a lack of research on the influence of green patents and environmental factors on green growth.

### The Relationship Between Green Technology and Green Growth

Earlier studies, e.g., Hongo's (20), Nassiry (21), Huang et al. (22), Azhgaliyeva et al. (23), considered production-based emissions and consumption-based emissions as proxies of green growth. In contrast, our model considers EAMFP as green growth. A number of previous studies, including Lee and Chou (24), Hao et al. (25), Wang et al. (1, 2), Yang et al. (26), and Fernandes et al. (27), support EAMFP as a green growth indicator for investigating the empirical model.Their arguments support the notion that green growth is strongly influenced by green technology in developed and developing economies.A few studies have examined the impact of green patents on green growth (28–30). Wang et al. (1, 2) investigated the impact of technological innovation (green patents) on green growth (e.g., EAMFP) by using the Bayer-Hanch Cointegration test for sample G7 countries. The authors analyzed that GDP and technological innovation (green patents) have remarkable impact on green growth in the long run. Furthermore, green growth primarily depends on technological innovation and GDP.

Another study, e.g., Nosheen et al. (31), estimated the impact of green technology on green growth for European Union countries by employing the cross-sectional dependence and Westerlund cointegration approach. The results demonstrated that green technology substantially increases green growth. However, outcomes indicate that other factors such as energy consumption and production-based technologies have a negative impact on green growth. Sohag et al. (32) also analyzed the association among green growth, energy, and technological innovation for sample OECD countries. Their findings concluded that there is a positive association between technological innovation and green growth in the long run, as estimated by the CS-ARDL approach. Furthermore, militarization is antagonistic to green growth. Ulucak (29) also researched the association between green technology and green growth by using sample emerging economies. The author found that green technology drastically upsurges green growth. For instance, patents (e.g., new technologies) reduce the harmful effects related to the environment and evade the use of natural resources in unsustainable development.

### Green Growth and the Environment

Besides, carbon emissions also have remarkable impacts on green growth. In order to support such assertion, Koondhar et al. (33) considered the agricultural production as a proxy of green growth in order to investigate the impact of carbon emissions. The authors examined the time-series data by employing the ARDL approach. The findings concluded that an increase in carbon emissions substantially reduce the green growth. In addition, unidirectional exists from green growth to carbon emissions. In contrast, Yang et al. (26) investigated the impact of green growth on carbon emissions. The authors also included the energy consumption in affecting the CO2 emissions. They argue green growth and energy consumption have substantial impact on carbon emissions in the long run. Moreover, there is unidirectional causal relationship from green growth and energy consumption to carbon emissions exist in the long run. Subsequently, Hao et al. (25) also debated that green growth has a remarkable impact on carbon emissions. It means that carbon emissions could be reduced due to improvement in EAMFP. In addition, Fernandes et al. (27) argued that green growth decouples natural resource use and environmental impacts from the continuation of economic growth. However, the Paris Agreement and the United Nations Sustainable Development Goals presume the continuation of economic growth (34, 35). In future prospects, continuous economic growth is not a substantial factor for sustainable use of natural resources. The reason behind this is that continuous economic growth substantially increases unsustainable production and consumption in high-GDP countries. Other studies, e.g., O'Neill (36), D'Alessandro et al. (37), Banerjee et al. (38), also examine whether economic growth has a remarkable impact on green growth. Furthermore, the initially positive trend in economic growth leads to increased green growth by utilizing natural resources. Thereafter, a continuation in economic growth declines the green growth concern over the sustainable use of natural resources. Hickel and Kallis (39) debate that continuation of economic expansion is not compatible with the global environment. However, absolute decoupling of GDP growth from natural resource use and carbon emissions is conceivable along with technological changes. In addition, Schreiner and Madlener (40) also found that green growth is substantially influenced by economic growth. However, their narrative reveals that macroeconomic growth improves green growth *via* key variables, e.g., income level, fiscal policies, and value added. Thus, the provision of such variables is a cope for green growth. In contrast, macroeconomic growth varies in each economy due to its diverse economic system (41–43).

### The Relationship Between Green Growth and Energy Consumption

Considering the outcome of energy consumption on green growth, a substantial number of studies found that energy consumption has a remarkable impact on green growth. However, energy consumption from fossil fuels adversely affects green growth, which leads to the deterioration of natural resources. In this context, Kirikkaleli and Adebayo (44) argued that renewable energy consumption increases green growth by reducing emissions in the atmosphere. Likewise, (45, 46) found that energy consumption incredibly impacts environmental quality. Furthermore, nonrenewable energy consumption deteriorates environmental quality because natural resources are being shrunk gradually with a rise in environmental pressure. Some upshots are also documented by numerous studies such as Baniya et al. (47), Sohag et al. (37), and Ulucak (29) for high-income countries concerning the relationship between energy consumption and green growth. Despite the negative effect of renewable energy consumption on green growth, studies, e.g., Farhani and Shahbaz (48) and Bulut (49), reported a positive effect of energy consumption (renewable and nonrenewable) on green growth. Lu et al. (50) also endorsed the positive effect of renewable energy on green growth.

This study fills the research gap by quantifying the impact of joint effect of environmental factors and green technology in green growth in high-GDP countries. The above literature lacks to give distinct explanations on the association with green growth. Thus, green technology is included in the current investigation. Also, the inclusion of joint effect of environmental factors plays a vital role in analyzing the influencers of green growth. More interestingly, previous studies merely considered the energy consumption excluding the fossil fuel. However, this study considers energy consumption from fossil fuel. The reason is that emissions are directly released from the fossil fuel used by different sectors of the economy. Furthermore, the current study assesses the role of linear, nonlinear, and joint effects of the GDP and environmental factors, which are ignored by previous studies in the literature.

## Methodology

### Theoretical Framework and Estimation Strategy

**Figure 1:**
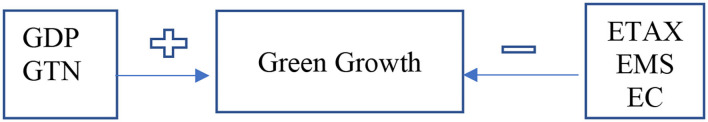
Theoretical framework.

We develop a theoretical framework based on the review of literature. Figure 1: The framework illustrates that green growth (GGR) is a function of GDP, energy consumption (ENC), ENTs, green technology (GTN), and emissions (EMS). Therefore, GDP may affect green growth through several economic activities such as trade, investment, agricultural and industrial productivity, etc. Economic activities require enormous natural resources, e.g., agricultural resources, which directly influence green development. More precisely, the environmental Kuznets curve (EKC) theory reveals that there are three effects, e.g., scale effect, composition effect, and technique effect. A scale effect suggests that an increase in GDP reduces green growth at an initial level because it requires more resources, such as natural resources (energy and raw materials). Besides, the composition effect reveals that the country's structural transformation from industrial to the service sector is anticipated to reduce the harmful consequences of economic growth on the environment. The technique effect shows that economies adopt advanced technologies along with growth in income, which makes progress in productivity related to the environment (51, 52).

Theoretical notion reveals that energy consumption is a critical factor for green growth. Green growth provides a wide-ranging concept of natural resources specific environmental services (OECD). It integrates the value of capital natural resources concerning the economic decisions for development planning. In addition, green growth also prevents the depletion of capital natural resource's value. However, energy consumption (EC) may affect the green growth (53–55).

### Model Specification

This study proposes the following model:


(1) $$\begin{array}{r} GGR_{it}=\beta_{0}+\beta_{1}\left(GDP_{it}\right)+\beta_{2}\left(ENC_{it}\right)+\beta_{3}\left(ENT_{it}\right)\\ \;+\beta_{4}\left(GTN_{it}\right)+\beta_{5}\left(EMS_{it}\right)+\varepsilon_{it} \end{array}$$

Where β′*s* indicate the slope of explanatory variables, and cross sections are denoted by “*i*”, (e.g., high-GDP countries), whereas, “*t*” show time period from 2000 to 2020. In addition, the terms: *GGR*, GDP, ENC, ENT, GTN, and EMS indicate green growth, GDP, energy consumption, ENTs, patents, and emissions.

The Equation (1) reveals that green growth is a function of GDP, ENC, ENT, GTN, and EMS. Previous arguments indicate that GDP has an expected positive or negative sign for green growth. It is anticipated that GDP has a positive sign $\left(\frac{GGR}{GDP}>0\right)$ or negative sign $\left(\frac{GGR}{GDP}<0\right)$. Similarly, energy consumption may also have a negative sign $\left(\frac{GGR}{ENC}<0\right)$. In addition, ENTs are anticipated to positively affect or negatively affect the green growth $\left(\frac{GGR}{ENT}>or<0\right)$. It is worth to mention that green technology has also expected positive effect on green growth $\left(\frac{GGR}{GTN}>0\right)$. Lastly, the emissions are anticipated to have negative effect on green growth $\left(\frac{GGR}{EMS}<0\;\right)$.


(2) $$\begin{array}{r} GGR_{it}=\beta_{0}+\beta_{1}\left(GDP_{it}\right)+\beta_{2}\left(GDP_{it}^{2}\right)+\beta_{3}\left(ENC_{it}\right)\\ \;+\beta_{4}\left(ENT_{it}\right)+\beta_{5}\left(GTN_{it}\right)+\beta_{6}\left(EMS_{it}\right)+\varepsilon_{it} \end{array}$$

Additionally, we extend Equation (1) by including the square of GDP (GDP^2^) to estimate the nonlinear effect on green growth. The square of GDP also has expected positive or negative effect on green growth $\left(\frac{GGT}{GDP2}>or<0\;\right)$.


(3) $$\begin{array}{l} GGR_{it}=\beta_{0}+\beta_{1}\left(GDP_{it}\right)+\beta_{2}\left(ENC_{it}\right)+\beta_{3}\left(ENT_{it}\right)\\ \;+\beta_{4}\left(GTN_{it}\right)+\beta_{5}\left(EMS_{it}\right)+\beta_{6}\left(EMS*ENC_{it}\right)+\varepsilon_{it} \end{array}$$

Besides, Equation (3) shows that the interaction term of emissions and energy consumption (EMS^*^ENC) is included in the based model in order to estimate the joint effect on green growth. Thus, the interaction term is also anticipated to have negative effect on green growth $\left(\frac{GGR}{EMS*ENC}>or<0\right)$.

### Operational Definition of the Variables

The green growth is defined as the level of natural assets that provide environmental services. Furthermore, it corresponds to the decoupling of economic growth from resources and environmental harmful impacts (56). Numerous researchers agree that green growth is a good and appropriate corridor for diverse economies and invoke it as a proper future route toward a low-carbon world (25, 57, 58). Conventionally, green growth is measured by EAMFP. Moreover, EAMFP also measures the residual growth in the joint production of desirable and undesirable items. Considering the influencers of GGR, GDP is the most important factor, defined as the value added of all commodities produced by the economy within a specific time period and measured in dollars. Energy consumption is also a crucial factor for green growth. It refers to the consumption of fossil fuels (coal, oil, and gas) by all sectors of the economy in millions of tons (59, 60). Subsequently, ENTs also effectively made significant changes in green growth. Therefore, it is defined as green taxes, ecotaxes, and pollution taxes, which are broad forms of governmental duties on entities (firms and companies) aimed at protecting the environment (45, 46, 61–63). Besides, patents also play a productive role in green growth. It indicates that the output of environmental innovation or new technologies can be used to combat environmental issues (64–66). In addition, emissions are also an important factor in influencing green growth. Crippa et al. (67); Yuping et al. (68) and Reisinger et al. (69) define it as the amount of substance released into the atmosphere. Table 1 provides the variable, measure, code, and source.

**Table 1:** Variables, measure, and source.

**Variable**	**Measure**	**Code**	**Source**
Green growth	Index of EAMFP	GGR	OECD
Gross domestic product	$US (million)	GDP	WDI
Energy consumption	Ton	ENC	WDI
Environmental taxes	% of GDP	ENT	OECD
Green technology	Number	GTN	OECD
Emissions	ton	EMS	OECD

*Author's derivation*.

### Data and Source

To estimate the effect of green technology, energy consumption, GDP, emissions, and ENTs on green growth, balanced panel data are used from period 2001 to 2020 for the top 20 high-GDP countries, namely USA, China, Japan, Germany, India, UK, France, Italy, Brazil, Canada, Russia, South Korea, Spain, Australia, Mexico, Indonesia, Netherlands, KSA, Turkey, and Switzerland. GGR, GTN, ENT, and EMS data are obtained from the website OECD, while GDP and ENC data are collected from the website of World Bank Indicator (WDI). Table 1 provides the variables, measure, and source. In addition, the descriptive statistics are reported in Table 2.

**Table 2:** Descriptive statistics.

**Variable**	**Obs**	**Mean**	**S.D**	**Min**.	**Max**.
GGR	400	0.926	1.326	−6.650	4.880
GDP	400	2.505	3.546	1.604	2.160
GTN	400	2.955	1.467	0.091	4.391
EC	400	3.887	3.775	0.001	4.389
ETN	400	2.205	2.094	0.100	16.976
EMS	400	14.311	1.336	11.707	18.071
EC	400	14.59	13.337	0.005	51.787

*Author's calculations*.

### Estimation Methods

#### Slope Homogeneity Test

Swamy (70) developed the framework to find if slope coefficients of the cointegration equation are homogeneous. Pesaran and Yamagata (71) improved Swamy's slope homogeneity test and formed two **“delta**” test statistics; $\tilde{\Delta }$ and ${\tilde{\Delta }}_{\text{a}d\text{j}}$.


$$\begin{array}{lll} \tilde{\Delta } & = & \sqrt{N}\left(\frac{N^{-1}\bar{S}-k}{\sqrt{2k}}\right)\sim X_{k}^{2}\\ {\tilde{\Delta }}_{adj} & = & \sqrt{N}\left(\frac{N^{-1}\bar{S}-k}{v\left(T,k\right)}\right)\sim N\left(0,1\right) \end{array}$$

Where, ***N*** denotes number of cross-section unit; ***S*** denotes the Swamy test statistic; ***k*** denotes independent variables. If ***p*** value of the test is larger than 5%, then the null hypothesis is accepted at a 5% significance level and the cointegrating coefficients are considered homogenous. $\tilde{\Delta }$ and ${\tilde{\Delta }}_{\text{a}d\text{j}}$ are suitable for large and small samples, respectively, where ${\tilde{\Delta }}_{\text{a}d\text{j}}$ is “mean-variance bias adjusted” version of $\tilde{\Delta }$. Therefore, standard delta test ($\tilde{\Delta }$) requires error not to be autocorrelated. By relaxing the assumptions of homoscedasticity and serial independence of (71, 72), developed a Heteroscedasticity and Autocorrelation Consistent (HAC) robust version of slope homogeneity test; **Δ**_**HAC**_ and (_**Δ**_**HAC**_ )**adj**_:


$$\begin{array}{l} \Delta_{HAC}=\sqrt{N}\left(\frac{N^{-1}S_{HAC}-k}{\sqrt{2k}}\right)\sim X_{k}^{2}\\ {\left(\Delta_{HAC}\right)}_{adj}=\sqrt{N}\left(\frac{N^{-1}S_{HAC}-k}{v\left(T,k\right)}\right)\sim N\left(0,1\right) \end{array}$$

The null hypothesis of slope homogeneity can be rejected in all cases because the probability values are smaller than 0.05 in all of the cases. The slope coefficients are not homogeneous. Heterogeneity exists across sample countries; we should employ heterogeneous panel techniques.

#### Cross-Sectional Dependence Tests

In order to detect the cross-dependency among the selected variables, the outcomes from cross-dependence (CD) test are reported in Table 3. Chudik and Pesaran (73) and Bailey, Kapetanios, and Pesaran (74) along with Bailey, Kapetanios, and Pesaran (75) versions of (76) CD tests are estimated to scrutinize the presence of cross-sectional dependence in residuals of estimable model. Both of the tests are statistically significant at 1%, supporting the assumption of cross-sectional dependence in the residuals of estimable model.

**Table 3:** Tests for cross-sectional dependence in residuals.

**Test**	**Statistic**	**Value**
$CD_{NT}^{2015}$	$\sqrt{\frac{2}{N\left(N-1\right)}}\overset{N-1}{\underset{i=1}{\sum }}\overset{N}{\underset{j=i+1}{\sum }}\frac{1}{\sqrt{T}}\;\overset{T}{\underset{t=1}{\sum }}\xi_{it}\xi_{j}$	6.194^a^
*CD*_*BKP*_	$\sqrt{\frac{TN\left(N-1\right)}{2}}{\hat{\bar{\rho }}}_{N}$	20.563^a^

a * represents statistical significance at 1%*.

*Authors' estimates*.

*Tests for Cross-Sectional Dependence in Residuals*

**Table:** 

$\text{C}{\text{D}}_{\text{N}\text{T}}^{2015}$	$\sqrt{\frac{2}{N\left(N-1\right)}}\overset{N-1}{\underset{i=1}{\sum }}\overset{N}{\underset{j=i+1}{\sum }}\frac{1}{\sqrt{T}}\overset{T}{\underset{t=1}{\sum }}\xi_{it}\xi_{j}$
**CD**_**BKP**_	$\sqrt{\frac{TN\left(N-1\right)}{2}}{\hat{\bar{\rho }}}_{N}$

Tests for Cross-Sectional Dependence in the Variables.


$$CD_{BKP}\;=\;\sqrt{\frac{TN\left(N-1\right)}{2}}{\hat{\bar{\rho }}}_{N}$$

Based on Bailey, Kapetanios, and Pesaran (74) and Bailey, Kapetanios, and Pesaran (75), Table 4 delves deeper by estimating the cross-sectional dependence statistic for relevant variables (*GGR*_*i, t*_, *GDP*_*i, t*_, *ENC*_*i, t*_, *ENT*_*i, t*_, *GTN*_*i, t*_ &* EMS*_*i, t*_). Most of the variables show statistically significant at 1%, showing cross-sectional dependence in the variables of estimable model.

**Table 4:** Tests for cross-sectional dependence in variables.

		**Value for:**					
**Test**	**Statistic**	**GGR_i, t_**	**GDP_i, t_**	**ENC_i, t_**	**ENT_i, t_**	**GTN_i, t_**	**EMS_i, t_**
*CD*_*BKP*_	$\sqrt{\frac{TN\left(N-1\right)}{2}}{\hat{\bar{\rho }}}_{N}$	19.300^a^	50.909^a^	1.024	2.800^a^	17.094^a^	−1.241

a * represents statistical significance at 1%*.

*Authors' estimates*.

#### Second Generation Unit Root Test

To test for stationarity, in the presence of cross-sectional dependence, we use second generation unit root tests. The cross-sectional dependence has a strong presence in residuals (and in variables) as shown in Table 5. It calls for checking stationarity using second generation of unit root tests since first generation of unit root tests (77, 78) do not account for cross-sectional dependence in testing for stationarity. Considering the evident cross-sectional dependence, we use second generation unit root tests proposed by Pesaran to shed light on the findings. Mathematically:


$$\begin{array}{l} \Delta y_{i,t}=a_{i}+b_{i}y_{i,t-1}+c_{i}{\bar{y}}_{t-1}+d_{i}{\bar{y}}_{t}+\varepsilon_{i,t} \end{array}$$

Where *a*_*i*_ is a deterministic term, ${\bar{y}}_{t}$ is the cross-sectional mean at time *t*, and ρ is the lag order. *t*_*i*_(*N, T*) denotes the corresponding *t* ratio of α_*i*_ and is known as cross-sectional Augmented Dickey Fuller (ADF) [CADF, attributed to Pesaran (79)]. The average of the *t* ratios gives the cross-sectional IPS (IPS) [cross sectional augmented IPS (CIPS), attributed to (80)]. In Table 6, these tests are estimated with a constant term at level and first difference. Mutual consensus, of both CADF and CIPS tests, reveals that all variables are stationary at first difference i.e. *I*(1).

**Table 5:** Second generation unit root tests for individual variables.

**Cross–Sectional ADF (CADF) test**											
GGR_i, t_	ΔGRD_i, t_	GDP_i, t_	ΔGDP_i, t_	ENC_i, t_	ENC_i, t_	ENT_i, t_	ENT_i, t_	GTN_i, t_	GTN_i, t_	EMS_i, t_	EMS_i, t_
−1.76	−3.22^a^	−0.98	−2.75^a^	−1.28	−4.74^a^	−1.80	−3.92^c^	−1.32	−3.60^a^	−1.99	−4.78^a^
**Cross–Sectional IPS (CIPS) test**											
GGR_i, t_	Δ*GRD_i, t_*	GDP_i, t_	Δ*GDP_i, t_*	ENC_i, t_	ENC_i, t_	ENT_i, t_	ENT_i, t_	GTN_i, t_	GTN_i, t_	EMS_i, t_	EMS_i, t_
−1.15	−2.07^c^	−1.25	−2.07^c^	−1.08	−2.99^a^	−1.78	−2.43^a^	−0.89	−2.28^a^	−1.27	−3.42^a^
GRD_i, t_ is I(1)		GDP_i, t_ is I(1)		ENC_i, t_ is I(1)		ENT_i, t_ is I(1)		PTN_i, t_ is I(1)		EMS_i, t_ is I(1)	

*By definition: $CIPS=\;\frac{\overset{N}{\underset{i=1}{\sum }}t_{i}\left(N,T\right)}{N}=\frac{\overset{N}{\underset{i=1}{\sum }}CADF_{i}}{N}$*.

*^a^ and ^c^ represent statistical significance at 1% and 10%, respectively*.

*Authors' estimates*.

**Table 6:** Slope homogeneity tests.

	**Statistic**	**Value**
(71 )		
$\tilde{\Delta }$	$\sqrt{N}\left(\frac{N^{-1}\bar{S}-k}{\sqrt{2k}}\right)\sim X_{k}^{2}$	5.873^a^
${\tilde{\Delta }}_{\text{a}d\text{j}}$	$\sqrt{N}\left(\frac{N^{-1}\bar{S}-k}{v\left(T,k\right)}\right)\sim N\left(0,1\right)$	7.019^a^
(72)
**Δ**_**HAC**_	$\sqrt{N}\left(\frac{N^{-1}S_{HAC}-k_{2}}{\sqrt{2k_{2}}}\right)$	5.723^a^
(_**Δ**_**HAC**_)**adj**_	$\sqrt{N}\left(\frac{N^{-1}{\bar{S}}_{HAC}-k_{2}}{v\left(T,k\right)}\right)\sim N\left(0,1\right)\;$	6.841^a^

a * represents statistical significance at 1%*.

*$\tilde{\Delta }$ and ${\tilde{\Delta }}_{\text{a}d\text{j}}$ represent the “simple” and “mean variance bias adjusted” slope homogeneity tests, respectively*.

***Δ**_**HAC**_ and (_**Δ**_**HAC**_)**adj**_ represent the “Heteroscedasticity and Autocorrelation Consistent” versions of “simple” and “mean variance bias adjusted” slope homogeneity tests, respectively*.

#### Augmented Autoregressive Distributed Lag

After checking the stationarity of the variables, we apply the CS-ARDL model. Attributed to (73), CS-ARDL is used to study the long-run and short-run relationship among *GGT*, *GDP*, *ENC*, *ENT*, *GTN*, and *EMS*. The equation is given as:


$$\begin{array}{l} D_{i,t}=\overset{p_{D}}{\underset{I=0}{\sum }}\vartheta_{I,i}D_{i,t-I}+\overset{p_{X}}{\underset{I=0}{\sum }}\delta_{I,i}X_{i,t-I}+\epsilon_{i,t} \end{array}$$

To solve the issue of cross-sectional dependency and slope heterogeneity, the extended version of the last equation is given as:


$$\begin{array}{l} D_{i,t}=\overset{p_{D}}{\underset{I=0}{\sum }}\vartheta_{I,i}W_{i,t-I}+\overset{p_{X}}{\underset{I=0}{\sum }}\delta_{I,i}X_{i,t-I}+\overset{p_{Z}}{\underset{I=0}{\sum }}\sigma_{i}^{{}^{\prime }}{I\bar{Z}}_{t-I}+\epsilon_{i,t} \end{array}$$

In the last equation, ${\bar{Z}}_{t-I}=\left({\bar{D}}_{i,t\bar{I}},{\bar{X}}_{i,t\bar{I}}\right)$ provides the averages, similarly lags are shown through *p*_*D*_, *p*_*X*_, *p*_*Z*_: *D*_*it*_ is dependent variable (here *GGT*), followed by *X*_*i, t*_ for all the independent variables (here *GDP*, *ENC*, *ENT*, *GTN*, and *EMS*. $\bar{Z}$ is dummy for time period. The long-run coefficients are generally represented as:


$$\begin{array}{l} {\hat{\theta }}_{CS-ARDL,i}=\frac{\sum_{I=0}^{p_{X}}{\hat{\delta }}_{I,i}}{1-\sum_{I=0}^{p_{D}}{\hat{\vartheta }}_{I,i}} \end{array}$$

Whereas, the following equation shows the mean group coefficients:


$$\begin{array}{l} {\hat{\bar{\theta }}}_{MG}=\frac{1}{N}\sum_{i=1}^{N}{\hat{\theta }}_{i} \end{array}$$

Similarly, the short-run coefficients are expressed with the following four equations:


$$\begin{array}{l} {\Delta D}_{i,t}=\vartheta_{i}\left[D_{i,t-1}-\theta_{i}X_{i,t}\right]-\overset{p_{D-1}}{\underset{I=1}{\sum }}\vartheta_{I,i},\Delta_{I}W_{i,t-I}\\ \;+\overset{p_{X}}{\underset{I=0}{\sum }}\delta_{I,i}\Delta_{I}X_{i,t}+\overset{p_{Z}}{\underset{I=0}{\sum }}\sigma_{i}^{{}^{\prime }}{I\bar{Z}}_{t}+\varepsilon_{i,t}\\ {\;\hat{\alpha }}_{i}=-\left(1-\overset{p_{D}}{\underset{I=1}{\sum }}{\hat{\vartheta }}_{I,i}\right)\\ \;{\hat{\theta }}_{i}=\frac{\sum_{I=0}^{p_{X}}{\hat{\delta }}_{I,i}}{{\hat{\alpha }}_{i}}\\ \;{\hat{\bar{\theta }}}_{MG}=\overset{N}{\underset{i=1}{\sum }}{\hat{\theta }}_{i} \end{array}$$

## Empirical Analysis and Discussion

### Descriptive Statistics

Table 2 reports the descriptive statistics on the selected variables. The results reveal that mean value and SD value of green growth (GGR) are 0.926 and 1.326 respectively, indicating narrow variation in observations from mean. The mean value of emissions (EMS), while SD value is 1.336, which means that observations vary within a narrow range over a period of time across the high-GDP countries. Most of the SD values are found lower, indicating that observation varies from the mean within a small range. The results also suggest that there is little variation in the energy consumption (EC) in the sample countries.

Table 3 provides the estimate of CD in residuals, the outcomes suggest that the existence of cross-dependence issue is validated. Furthermore, in order to check the consistent outcomes, we detect the CD issues in each variable for the sample countries. Table 4 reports the summary of the CD test, which indicates that selected variables have CD issues. Moreover, absolute value ranging from −1.241 to 50.909 confirm the CD. This suggests that high-GDP countries are dependent to each other concerning green growth, green technological, and environmental factors within the time period. Besides, the variables of high-GDP countries can influence the other countries by any shock with regards to CD issues. The outcomes are reported in Table 6, which unveils that heterogeneity exists in their slope coefficients. Moreover, high-GDP countries panel contains varying rates of growth level. Additionally, Table 5 provides the outcomes of CIPS and CADF unit root test. Some variables, e.g., GGR, GDP, EC, EMS, and GTN, indicate the nonstationarity at the level, while most of the variables show complete at first difference for CADF. However, the outcomes from CIPS show that variables are statistically significant at first difference.

### Estimating the Long-Run and Short-Run Relationship

After the evaluation of cross-dependence among the variables, we estimate the long-run and short-run relationship between green growth and its influencers by using CS-ARDL approach. Therefore, Table 7 reports the outcomes of CS-ARDL estimation. The results reveal that GDP, energy consumption, ENTs, patents, and emissions have statistically significant relationship with green growth. The positive values in the short and long run (CS-ARDL) of coefficient of GDP and patents show that the increase in these variables helps green growth in sample countries. i.e., $\frac{\partial GGR_{i,t}}{\partial GDP_{i,t}}>0$ and $\frac{\partial GGR_{i,t}}{\partial PTN_{i,t}}>0$. On the other hand, negative values in the short and long run (CS-ARDL) of coefficients of energy consumption, ENTs, and emissions suggest that if there is increase in these variables there will be decrease in green growth in sample countries. i.e., $\frac{\partial GGR_{i,t}}{\partial ENC_{i,t}}<0$, $\frac{\partial GGR_{i,t}}{\partial ENT_{i,t}}<0$ and $\frac{\partial GGRT_{i,t}}{\partial EMS_{i,t}}<0$. More analytically, a 1% increase in GDP upsurges GGR to around 42.4% and 33.8% in the long run and short run, respectively.

**Table 7:** CS–ARDL estimations.

	**Dependent variable:** **GGR**_****i**, **t****_				
	**Long run**			**Short run**	
**Variable**	**Slope coefficient**	**Standard errors**	**Variable**	**Slope coefficient**	**Standard errors**
*GDP*_*i, t*_	0.424^a^	0.125	Δ*GDP*_*i, t*_	0.338^a^	0.152
*ENC*_*i, t*_	−0.642^c^	0.344	Δ*ENC*_*i, t*_	−1.571^c^	0.877
*ENT*_*i, t*_	−1.611^b^	0.625	Δ*ENT*_*i, t*_	−0.542^b^	0.286
*GTN*_*i, t*_	0.138^b^	0.054	Δ*GTN*_*i, t*_	1.316^b^	0.602
*EMS*_*i, t*_	−0.043^b^	0.018	Δ*EMS*_*i, t*_	−1.134^b^	0.565
–	–	–	*ECT*(−1)	−0.079^a^	0.027

*^**a**^, ^**b**^, and ^**c**^ show statistical significance at 1, 5, and 10%, respectively*.

*Authors' estimations*.

The reason is that high-GDP countries spend larger amount on economic activities, particularly sustainable development, which aimed to protect the environment by diminishing the undesirable outputs. Consequently, green growth is improved in progressive inclination. This finding can be a supportive evidence for the findings of Fernandes et al. (27), Savin et al. (81), and Hao et al. (25). Concerning the effect of energy consumption (EC), the coefficient magnitude indicates that a 64.2% decrease in GGRT is due to energy consumption in the long run. Moreover, in the short-run, a 175.1% decrease in green growth is due to EC. It is worth mentioning that energy consumption from fossil fuel (coal, oil, and gas) has an adverse effect on green growth, excluding electricity or hybrid and biomass (82, 83).

Besides, the progressive effect of green patents on green growth is shown in current analysis for the OECD countries. It implies that a 13.8% increase in green growth is due to a 1% change in GTN in the long run. On the contrary, the short-run outcomes show that a 1% change in GTN upsurges 131.6% green growth. Former studies, e.g., Fernandes et al. (27), Urbaniec et al. (84), and Wang et al. (1, 2), also confirm our findings over positive association between green patents and green growth. Therefore, the results demonstrate that green patents may affect in distinctive perspective: for instance, reduce the harmful effects on environment, expansion in agro-industrial output, preserve the natural resources, and augment the capital accumulation in high-income countries. The reasons behind those high-income countries are enriched wide-ranging economic activities, which cause the deterioration of nature-based assets and release the greenhouse emissions. Simultaneously, these countries have the potential to utilize the resources to preserve the natural resources, increase green growth, and protect the environment (31).

In addition, emissions have negative impact on green growth. It implies that a 4.3% decrease in green growth is due to a 1% change in emissions in the long run. On the contrary, the short-run outcomes also validate that there is a negative correlation between GGR and EMS. In contrast, Hao et al. (25) argue that an increase in green growth reduces the emissions in the environment, because EAMFP (e.g., green growth) drastically preserve the natural resources and impede the greenhouse emissions. Thus, our findings contradict that emissions reduce the green growth (e.g., EAMFP) through agriculture and industrial framework. Besides, the results for *ECM*(−1) show that around 7.9% disequilibrium is corrected every year.

Considering the nonlinear effect of GDP, the coefficient of GDP^2^ has a negative impact on green growth in the sample countries, e.g., $\frac{\partial GGR_{i,t}}{\partial {GDP2}_{i,t}}<0$, as shown in Table 8. It is an indication of u-shaped relationship between GGT and GDP. More analytically, an increase in GDP upsurges the green growth (e.g., EAMFP) at initial level. It means that countries require enormous nature-based assets for economic activities. Under such circumstance, green growth has a tendency to increase through nature-based resource augmentation. However, after reaching a specific level, green growth began to decline with a rise in GDP due to higher aggregate demand, which shortens the supply of nature-based assets. The results demonstrate that a 1% increase in GDP^2^ reduces 22.1% green growth in the long run. On the contrary, the short-run results also validate that a 33% decrease in green growth is due to a 1% change GDP^2^.

**Table 8:** CS–ARDL estimations.

	**Dependent variable:** **GGR**_****i**, **t****_						
	**Long run**				**Short run**		
**Variable**	**Linear**	**Non–Linear**	**Interaction**		**Linear**	**Non–Linear**	**Interaction**
*GDP*_*i, t*_	0.424^a^ (0.125)	0.471^a^ (0.131)	0.527^b^ (0.260)	Δ*GDP*_*i, t*_	0.338^a^ (0.152)	0.347^b^ (0.134)	0.107^a^ (0.035)
$GDP_{i,t}^{2}$	–	−0.221^c^ (0.130)	–	$\Delta GDP_{i,t}^{2}$	–	−0.330^c^ (0.192)	–
*ENC*_*i, t*_	−0.642^c^ (0.344)	−0.531^b^ (0.231)	−0.443^c^ (0.252)	Δ*ENC*_*i, t*_	−1.571^c^ (0.877)	−0.104^a^ (0.032)	−0.267^c^ (0.140)
*ENT*_*i, t*_	−1.611^b^ (0.625)	−0.169^b^ (0.077)	−0.429^a^ (0.138)	Δ*ENT*_*i, t*_	−0.542^b^ (0.286)	−0.648^b^ (0.295)	−0.345^b^ (0.134)
*GTN*_*i, t*_	0.138^b^ (0.054)	0.478^a^ (0.144)	0.658^b^ (0.310)	Δ*GTN*_*i, t*_	1.316^b^ (0.602)	0.655^c^ (0.352)	0.453^b^ (0.181)
*EMS*_*i, t*_	−0.043^b^ (0.018)	−0.191^b^ (0.089)	−0.966^c^ (0.499)	Δ*EMS*_*i, t*_	−1.134^b^ (0.565)	−1.160^c^ (0.606)	−2.624^a^ (0.558)
$\left(\begin{array}{c} ENC_{i,t}\\ \times EMS_{i,t} \end{array}\right)$	–	–	−0.261^c^ (0.134)	$\Delta \left(\begin{array}{c} ENC_{i,t}\\ \times EMS_{i,t} \end{array}\right)$	–	–	−0.633^b^ (0.286)
–	–	–	–	*ECT*(−1)	−0.079^a^ (0.027)	−0.1907^b^ (0.089)	−0.253^a^ (0.060)

*^**a**^, ^**b**^, and ^**c**^ show statistical significance at 1%, 5% and 10%, respectively*.

*Values in parenthesis are standard errors*.

*Authors' estimations*.

Concerning over the joint effect of energy consumption and emissions (EC^*^EMS), the coefficient magnitude of EC^*^EMS is negative and statistically significant in the sample countries e.g., $\frac{\partial GGR_{i,t}}{\partial {EC^{*}EMS}_{i,t}}<0$. It implies that a 26.1% decrease in green growth is due to a 1% change in interaction term of energy consumption and emissions in the long run. The short-run results also confirm that a 1 % change in interaction term of EC^*^EMS reduces the 63.3% green growth. Thus, there is a negative correlation among the interaction of EC, EMS, and GGR.

### Robustness Check Using CCEMG and AMG Estimators

To check the robustness of the results obtained by CS-ARDL, we deploy two additional techniques that cater cross-sectional dependence. Pesaran (85) forwarded Common Correlated Effects Mean Group (CCEMG) model with estimator β_*j*_(= β+ω_*j*_), which implies a common parameter β across the countries while ω_*j*_~*IID*(0, *V*_ω_). CCEMG has the tendency to asymptotically eliminate CD. Moreover, it allows heterogeneous slope coefficients across group members, which are captured simply by taking the average of each country's coefficient.

Attributed to (86), augmented mean group (AMG) is a surrogate to CCEMG, which also captures the unobserved common effect in the model. Moreover, AMG estimator also measures the group-specific estimator and takes a simple average across the panel. The highlight of Augmented Mean Group (AMG) is that it follows first difference Ordinary Least Squares (OLS) for pooled data and is augmented with year dummies.

The estimable model can be written as follows:


$$\begin{array}{l} {GGR}_{it}=\alpha_{i}+c_{i}t+d_{i}{\hat{\mu }}_{t}^{\upsilon a\Delta }+\beta_{i,1}\left({GDP}_{i,t}\right)+\beta_{i,2}\left({EC}_{i,t}\right)\\ \;+\beta_{i,3}\left({ET}_{i,t}\right)+\beta_{i,4}\left({GTN}_{i,t}\right)+\beta_{i,5}\left({EMS}_{i,t}\right)+\varepsilon_{i,t} \end{array}$$

where, ***i*** stands for cross-sectional dimension ***i*=1,…,*n*** and time period ***t*=1,…,*t*** and **α**_***i***_ represents country specific effects and ***d***_***i***_***t*** denotes heterogeneous country specific deterministic trends. α_*i*_ is related with the coefficient of respective independent variables $\beta_{i1}=\frac{\alpha_{i1}}{1-\alpha_{i1}}$, $\beta_{i2}=\frac{\alpha_{i2}}{1-\alpha_{i2}},\;$and $\beta_{i2}=\frac{\alpha_{i2}}{1-\alpha_{i2}}\;$ that are considered as heterogeneous across the countries. It is also assumed that the short run dynamics and their adjustment toward long run take place *via* error term $u_{i,t}\left(={\overset{´}{\Gamma }}_{i}f_{t}+\varepsilon_{i,t}\right)$. *f*_*t*_ characterizes the vector of unobserved common shocks. *f*_*t*_ can be either stationary or nonstationary, which does not influence the validity of the estimation (87). AMG estimation finds an explicit estimate for *f*_*t*_, which renders ${\hat{\mu }}_{t}^{\upsilon a}$ (common dynamic process) economic meaningfulness. Total factor productivity (TFP) is one of the plausible interpretations of ${\hat{\mu }}_{t}^{\upsilon a}$. Its coefficient *d*_*i*_ represents the implicit factor loading on common TFP. In addition, the cross-sectional specific errors ε_*i, t*_ are permissible to be serially correlated over time and weakly dependent across the countries (88). However, the regressors and unobserved common factor have to be identically distributed.

Table 9 reports estimate, yield positive relationship between *GGR* and *GDP*. According to CCEMG estimates, positive and significant relationship exists between GGR and GDP, i.e. ${\left(\beta_{GDP}^{CCE}\right)}_{1\%}=0.275$. Similarly, using AMG estimation technique, GGR and GDP show a positive relationship, i.e. ${\left(\beta_{GDP}^{AMG}\right)}_{1\%}=0.564$. Moreover, the slope parameters for energy consumption are negative for the two estimation techniques, i.e. ${\left(\beta_{ENC}^{CCE}\right)}_{10\%}=-0.675$ and ${\left(\beta_{ENC}^{AMG}\right)}_{10\%}=-1.138$, respectively. In addition, the slope parameters for ENTs are ${\left(\beta_{ENT}^{CCE}\right)}_{5\%}=-0.226$ and ${\left(\beta_{ENT}^{AMG}\right)}_{5\%}=-0.033$, respectively. Relationship of green technology with green growth is evident through their slope parameters, i.e. ${\left(\beta_{GTN}^{CCE}\right)}_{5\%}=0.334$ and ${\left(\beta_{GTN}^{AMG}\right)}_{5\%}=0.044$, respectively. Furthermore, the slope parameters for emissions are ${\left(\beta_{EMS}^{CCE}\right)}_{5\%}=-1.541$ and ${\left(\beta_{EMS}^{AMG}\right)}_{5\%}=-1.311$, respectively. The model has withstood the robustness check, as the additional estimations are done using CCEMG and AMG techniques. These results are in lines with that of CS-ARDL estimates. Signs are unchanged, both in CCEMG and AMG, while statistical significance is between 1% and 10%. The Common Dynamic Process (*CDP*) is also significant at 1%, ${\hat{\mu }}_{t}^{\upsilon a}=0.859$. It essentially means that the role of some unobserved variables such as regional and international agreements, common policies toward other countries, and technological diffusion across countries also have a positive effect on green growth.

**Table 9:** CCEMG and AMG estimations.

	**Dependent variable:** **GGR**_****i**, **t****_			
	**CCEMG**		**AMG**	
**Variable**	**Slope coefficient**	**Standard errors**	**Slope coefficient**	**Standard errors**
*GDP*_*i, t*_	0.275^a^	0.133	0.569^a^	0.054
*ENC*_*i, t*_	−0.675^c^	0.278	−1.138^c^	0.479
*ENT*_*i, t*_	−0.226^b^	0.124	−0.033^b^	0.020
*GTN*_*i, t*_	0.334^b^	0.173	0.044^b^	0.015
*EMS*_*i, t*_	−1.541^b^	0.241	−1.311^b^	0.656
*CDP*	–	–	0.859^a^	0.196
Country Trend	−0.012	0.037	−0.005	0.005
Constant	0.4498	0.396	0.938^a^	0.228

*^a^, ^b^, and ^c^ show statistical significance at 1, 5, and 10%, respectively*.

*Author's estimations*.

### What Causes What?

#### Panel Granger Causality Test

Since green growth relationship is empirically established, it is important to know the cause-effect role in this relationship. Following statistical tools shall help us in furnishing it. Work of (89) laid the foundation of causality test that uses the bivariate regressions in a panel data context:


$$\begin{array}{l} y_{i,t}=\alpha_{0,i}+\alpha_{1,i}\;y_{i,t-1}+\ldots +\alpha_{p,i}\;y_{i,t-p}+\beta_{1,i}\;x_{i,t-1}+\ldots \\ \;+\beta_{p,i}\;x_{i,t-p}+\epsilon_{i,t}\\ x_{j,t}=\alpha_{0,j}+\alpha_{1,j}\;x_{j,t-1}+\ldots +\alpha_{p,j}\;y_{j,t-p}+\beta_{1,j}\;y_{j,t-1}+\ldots \\ \;+\beta_{p,j}\;y_{j,t-p}+\varepsilon_{j,t} \end{array}$$

Depending on the assumptions about homogeneity of the coefficients across cross-sections, there are two forms of panel causality test. First and conventional type treats the panel data as one large stacked set of data and performs the causality test in the standard way, that assumes all coefficients same across all cross-sections.


$$\begin{array}{l} \alpha_{0,i}=\alpha_{0,j},\;\alpha_{1,i}=\alpha_{1,j},\ldots ,\alpha_{p,i}=\alpha_{p,i},\;\forall_{i,j}\\ \beta_{1,i}=\beta_{1,j},\ldots ,\beta_{p,i}=\beta_{p,i},\;\forall_{i,j} \end{array}$$

Results of panel Granger causality are shown in Table 10.

**Table 10:** Panel granger causality test results.

**Causality**	****F**-Stat**	****p**-value**	**Remarks**
*GDP*_*i, t*_ **→** *GGR*_*i, t*_	8.119	0.000	Bi–causal Relationship between Green Growth and Gross Domesetic Product.
*GGR*_*i, t*_→*GDP*_*i, t*_	5.852	0.003	
*EMS*_*i, t*_→*GGR*_*i, t*_	4.321	0.002	Bi–causal Relationship between Green Growth and Emissions
*GGR*_*i, t*_→*EMS*_*i, t*_	3.212	0.000	
*EC*_*i, t*_→*GGR*_*i, t*_	3.654	0.006	Bi–causal Relationship between Green Growth and Energy Consumption
*GGR*_*i, t*_→*EC*_*i, t*_	2.544	0.000	
*GTN*_*i, t*_→*GGR*_*i, t*_	2.344	0.154	Ui–causal Relationship between Green Growth and Green Technology
*GGR*_*i, t*_→*GTN*_*i, t*_	4.543	0.000	

*Authors' estimates*.

Bi-causality between GDP (*GDP*_*i, t*_), EMS (*EMS*_*i, t*_), EC(*EC*_*i, t*_), and Green Growth (*GGR*_*i, t*_) are evident from results in Table 10 as both statistics are statistically significant at 1%. Any policy shock in GGR may affect GDP. More precisely, green growth may affect the GDP though policies implications by the governmental bodies. It means that policies over EAMFP can made drastic changes in GDP. Comparably, bi-causality between EMS and GGR is also evident that any policy shock in emissions may directly affect the green growth. In addition, bi-causality between energy consumption (*EC*_*i, t*_) and green growth (*GGR*_*i, t*_) also shows that any policy in energy consumption may affect the green growth. The reason behind that green growth is also influenced by the energy consumption (fossil fuels-coal, oil, and gas), which was discovered from nature-based assets.

#### Rationale for Dumitrescu-Hurlin Causality

This article, primarily, uses the conventional type of Granger causality. However, one of the main issues specific to panel data models refers to the specification of the heterogeneity between cross-sections. To consider the heterogeneity across cross-sections, Dumitrescu-Hurlin (90) made an assumption of allowing all coefficients to be different across cross-sections. In this causality context, the heterogeneity can be between the heterogeneity of the regression model and/or in terms of causal relationship from *GDP, EC, EMS* to *GGR*. Indeed, the model considered may be different from an individual to another, whereas there is a causal relationship from *GDP, EC, EMS* to *GGR* for all individuals.

Table 11 shows statistical significance of ${\tilde{Z}}_{N}^{HNC}$ test statistic, which shows that null hypothesis can be rejected, that is, *GDP*_*i, t*_, *EMS*_*i, t*_, and *EC*_*i, t*_ do not homogeneously cause *GGR*_*i, t*_. Same holds for the null hypothesis that *GGR*_*i, t*_ do not homogeneously cause *GDP*_*i, t*_, *EMS*_*i, t*_, *and EC*_*i, t*_. Therefore, it can be inferred that a bi-causal relationship exists between GDP and GGR. This specialized form of causality provides the insights into the causal relationship without contradicting the primary result of bi-causal Granger causality in Table 10. “Homogenous causality” can be attributed to “universal concern” for sustainable development and hence green economy.

**Table 11:** Dumitrescu–Hurlin causality test results.

**Causality**	** ${\text{W}}_{\text{N},\text{T}}^{\text{H}N\text{C}}$ **	** ${\tilde{\text{Z}}}_{\text{N}}^{\text{H}N\text{C}}$ **	**p–value**	**Remarks**
*GDP*_*i, t*_→*GGR*_*i, t*_	1.977	2.066	0.039	Homogeneous Bi–causal Relationship between Green Growth and Gross Domesetic Product.
*GGR*_*i, t*_→*GDP*_*i, t*_	2.291	2.842	0.005	
*EMS*_*i, t*_→*GGR*_*i, t*_	1.321	1.983	0.000	Homogeneous Bi–causal Relationship between Green Growth and Emissions
*GGR*_*i, t*_→*EMS*_*i, t*_	1.432	1.735	0.002	
*EC*_*i, t*_→*GGR*_*i, t*_	2.432	1.876	0.032	Homogeneous Bi–causal Relationship between Green Growth and Energy Consumption
*GGR*_*i, t*_→*EC*_*i, t*_	1.987	1.343	0.043	
*GTN*_*i, t*_→*GGR*_*i, t*_	2.134	1.039	0.136	Homogeneous Ui–causal Relationship between Green Growth and Green Technology
*GGR*_*i, t*_→*GTN*_*i, t*_	1.432	1.234	0.001	

## Conclusions

This study investigates the effects of green technology and environmental factors on green growth for high-GDP countries. For this purpose, we choose twenty high-GDP countries as a sample size period from 2000 to 2020. Second-generation models such as (71, 76) are used to test the cross-dependence and slope heterogeneity, respectively. In addition, this study employed the unit roots test (CIPS and CADF) of (80). The outcomes suggest that the model has CD and slope heterogeneity issues. Moreover, the findings from CS-ARDL test reveal that GDP upsurges green growth in the long run and short run for high-GDP countries. In contrast, the square of GDP deteriorates the green growth, because of excess use of natural resources. The estimated coefficient of energy consumption reveals that green growth is substantially declined by the use of energy consumption, particularly fossil fuel (oil, coal, and gas). Besides, emissions have negative impact on green growth in the sample countries.

More interestingly, ENTs have also negative impact on green growth. The reason may behind that imposition of aggressive taxes on environmental institutions failed to increase the green growth. The findings also suggest that the green technology has positive impact on green growth in the long run and short run. The joint effect of energy consumption and emissions reveals that emissions reduce the green growth through energy consumption, which comes from fossil fuel. Finally, Dumitrescu and Hurlin (D&H) causality outcomes suggest that any policy to target gross domestic product (GDP), energy consumption (EC), and emissions (EMS) significantly changes growth (GGR) and *vice versa*. On the contrary, any policy related to green technology significantly affects the GGT, while policy change in GGT does not affect the GTN.

## Policy Implications

Based on empirical findings, this study recommends some policy implications: (1) the findings highlight that continuous economic growth in high-GPD countries is not favorable to green growth. However, it is noteworthy that linear effect of economic growth is positive. The policymakers should consider the economic planning concern over green growth. (2) High-GDP countries should adopt alternative strategies of energy consumption such as fossil fuels (oil, coal, and gas). More precisely, countries must reduce the consumption level of energy in order to increase the green growth that may protect ecological environment. (3) Policymakers should focus on green technology by innovating the new methods or products related to environmental, which directly enhance the green growth. (4) High-GDP countries should manage their economic activities in order to reduce the emissions that pollute the environment and decrease the green growth. (5) Countries should avoid the imposition of aggressive ENTs on environmental institutions. On the contrary, the progressive ENTs should be imposed on environmental institutions, because such taxes enhance the green growth.

The scope of this study is limited to high-GDP countries and only a few variables are considered in the model. This study emphasizes on energy consumption that comes from fossil fuel such as oil, coal, and gas. Likewise, it also considers the patent related to environment. Furthermore, total emissions from all sector of the economy are included. Lastly, ENTs are considered to be analyzed. Thus, future studies can be extending the model by including economic complexity, green financing, and industrialization. Besides, the study can be assessed by covering the geographical areas such as BRICS, OECD, EU, and OBOR.

## Data Availability Statement

The datasets presented in this study can be found in online repositories. The names of the repository/repositories and accession number(s) can be found here: stats.oecd.org.

## Author Contributions

ZH conceived the concept, developed the model and analyzed. BM employed the estimation models. MK and RT critically discussed and reviewed the draft to accomplishment.

## Conflict of Interest

The authors declare that the research was conducted in the absence of any commercial or financial relationships that could be construed as a potential conflict of interest.

## Publisher's Note

All claims expressed in this article are solely those of the authors and do not necessarily represent those of their affiliated organizations, or those of the publisher, the editors and the reviewers. Any product that may be evaluated in this article, or claim that may be made by its manufacturer, is not guaranteed or endorsed by the publisher.

## References

[1] Wang Y Li Y Zhu Z Dong J. Evaluation of green growth efficiency of oil and gas resource-based cities in China. Clean Techn Environ Policy. (2021) 23:1785–95. 10.1007/s10098-021-02060-9

[2] Wang KH Umar M Akram R Caglar E. Is technological innovation making world “Greener?” An evidence from changing growth story of China. Technol Forecast Soc Change. (2021) 165:120516. 10.1016/j.techfore.2020.120516

[3] IPCC. Climate Change 2014: Synthesis Report. Contribution of Working Groups I, II and III to the Fifth Assessment Report of the Intergovernmental Panel on Climate Change (Geneva: IPCC). (2014). p.151.

[4] Kasecker TP Ramos-Neto MB da Silva JMC Scarano FR. Ecosystem-based adaptation to climate change: defining hotspot municipalities for policy design and implementation in Brazil. Mitig Adapt Strateg Glob Chang. (2018) 23:981–93.

[5] OCED. Towards Green Growth: Monitoring Progress. (2020). Available online at: https://www.oecd-ilibrary.org/environment/towards-green-growth-monitoring-progress_9789264111356-en

[6] Chen C Lan Q Gao M Sun Y. Green total factor productivity growth and its determinants in China's industrial economy. Sustainability. (2018) 10:1052–4.

[7] Cao P Bai YP. The temporal and spatial pattern of green development efficiency in China and its influencing factors. Gansu Soc Sci. (2018) 4:242–8.

[8] Tian Z Wei XY Ding XH. Evaluation of China's regional industry green development index and analysis of its influencing factors. Ecol Econ. (2018) 34:103–8.

[9] Dong XJ Shi T. The green development efficiency and influencing factors of central plains urban agglomeration. Reg Econ Rev. (2018) 5:116–22.

[10] Jason H Giorgos K. Is Green Growth Possible? New Political *Economy*. (2020) 25:469–86. 10.1080/13563467.2019.159896412291328

[11] Yuying Y Haixiang G Linfei C Xiao L Mingyun Gu Xiaoling K. Regional analysis of the green development level differences in Chinese mineral resource-based cities. Resour Policy. (2019) 61:261–72.

[12] Nutman AP Friend CR Horie K Hidaka H. The Itsaq Gneiss Complex of southern West Greenland and the construction of Eoarchaean crust at convergent plate boundaries. Develop Precambrian Geol. (2007) 15:187–218.

[13] Apergis N Payne JE. Renewable energy consumption and growth in Eurasia. Energy Econ. (2010) 32:1392–7. 10.1016/j.eneco.2010.06.001

[14] Shahbaz M Nasir MA Roubaud D. Environmental degradation in France: the effects of FDI, financial development, and energy innovations. Energy Econ. (2018) 74:843–57. 10.1016/j.eneco.2018.07.020

[15] Sandberg M Klockars K Wilen K. Green growth or degrowth? assessing the normative justifications for environmental sustainability and economic growth through critical social theory. J Clean Prod. (2019) 206:133–41. 10.1016/j.jclepro.2018.09.175

[16] Hussain Z Miao C Zhang W Khan MK Xia Z. Assessing the Role of Environmental Expenditures and Green Transport in Emissions Released by Transport: An Application of ARDL Approach. Front Environ Sci. (2021) 9:769608. 10.3389/fenvs.2021.769608

[17] Bhattacharya M Paramati SR Ozturk I Bhattacharya S. The effect of renewable energy consumption on economic growth: evidence from top 38 countries. Appl Energy. (2016) 162:733–41. 10.1016/j.apenergy.2015.10.104

[18] Inglesi-Lotz R Dogan E. The role of renewable versus non-renewable energy to the level of CO2 emissions a panel analysis of sub-Saharan Africa's Big 10 electricity generators. Renew Energy. (2018) 123:36–43. 10.1016/j.renene.2018.02.041

[19] Mensah CN Long X Boamah KB Bediako IA Dauda L Salman M. The effect of innovation on CO 2 emissions of OCED countries from 1990 to 2014. Environ Sci Pollut Res. (2018) 25:29678–98. 10.1007/s11356-018-2968-0 30144011

[20] Hongo H. Renewable and green energy resources technologies: lessons learnt in Sub-Saharan Africa (SSA). Renew Energy Sources Their Appl. (2013) 17.

[21] Nassiry D. The role of fintech in unlocking green finance: Policy insights for developing countries. ADBI Working Paper Series. (2018). 10.1007/978-981-13-0227-5_27

[22] Huang B Punzi MT Wu Y. Do Banks Price Environmental Risk? Evidence From a Quasi Natural Experiment in the People's Republic of China. ADBI Working Paper Series. Tokyo: Asian Development Bank Institute. (2019). 10.2139/ssrn.3541472

[23] Azhgaliyeva D Kapsaplyamova Z Low L. Implications of fiscal and financial policies for unlocking green finance and green investment. ADBI Working Paper Series. (2018). 10.1007/978-981-13-0227-5_32

[24] Lee CM Chou HH. Green growth in Taiwan—An application of the oecd green growth monitoring indicators. The Singapore Economic Review. (2018) 63:249–74. 10.1142/S0217590817400100

[25] Hao LN Umar M Khan Z Ali W. Green growth and low carbon emission in G7 countries: how critical the network of environmental taxes renewable energy and human capital is? Science of The Total Environment. (2021) 752:141853. 10.1016/j.scitotenv.2020.141853 32889278

[26] Yang H Shahzadi I Hussain M. USA carbon neutrality target: Evaluating the role of environmentally adjusted multifactor productivity growth in limiting carbon emissions. J Environ Manage. (2021) 298:113385. 10.1016/j.jenvman.2021.113385 34371219

[27] Fernandes CI Veiga PM Ferreira JJ Hughes M. Green growth versus economic growth: Do sustainable technology transfer and innovations lead to an imperfect choice? Business Strategy and the Environment. (2021) 30:2021–37. 10.1002/bse.273025855820

[28] Popp D. Innovation and climate policy. NBER. (2010). 10.3386/w15673

[29] Ulucak DR. How do environmental technologies affect green growth? evidence from BRICS economies. Sci Total Environ. (2020) 712:136504. 10.1016/j.scitotenv.2020.136504 31931216

[30] Khan Z Ali S Umar M Kirikkaleli D Jiao Z. Consumptionbased carbon emissions and international trade in G7 countries: The role of environmental innovation and renewable energy. Sci. Total Environ. (2020) 138945. 10.1016/j.scitotenv.2020.138945 32416502

[31] Nosheen M Iqbal J Abbasi MA. Do technological innovations promote green growth in the European Union? Environmental Science and Pollution Research. (2021) 28:21717–29. 10.1007/s11356-020-11926-2 33415613

[32] Sohag K Husain S Hammoudeh S Omar N. Innovation, militarization, and renewable energy and green growth in OECD countries. Environ Sci Pollut Res. (2021) 28:36004–17. 10.1007/s11356-021-13326-6 33686598

[33] Koondhar MA Aziz N Tan Z Yang S Abbasi KR Kong R. Green growth of cereal food production under the constraints of agricultural carbon emissions: a new insights from ARDL and VECM models. Sustain Energy Technol Assess. (2021) 47:101452. 10.1016/j.seta.2021.101452

[34] Alexander S. Sustained economic growth: United Nations mistake the poison for the cure. Australian Options. (2015) 18:19–22. 10.3316/informit.15711165787473925677225

[35] Hickel J. The Paris climate deal won't save us–our future depends on de-growth. The Guardian. (2017) 3.

[36] O'Neill DW. Beyond green growth. Nat Sustain. (2020) 3:260–1. 10.1038/s41893-020-0499-4

[37] D'Alessandro S Cieplinski A Distefano T Dittmer K. Feasible alternatives to green growth. Nat Sustain. (2020) 3:329–35. 10.1038/s41893-020-0484-y

[38] Banerjee O Bagstad KJ Cicowiez M Dudek S Horridge M Alavalapati JR. Economic, land use, and ecosystem services impacts of Rwanda's Green Growth Strategy: An application of the IEEM+ ESM platform. Science of The Total Environment. (2020) 729:138779. 10.1016/j.scitotenv.2020.138779 32380323

[39] Hickel J Kallis G. Is green growth possible? New Political Econ. (2002) 25:469–86.

[40] Schreiner L Madlener R. A pathway to green growth? Macroeconomic impacts of power grid infrastructure investments in Germany. Energy Policy. (2021) 156:112289. 10.1016/j.enpol.2021.112289

[41] Song M., Wang, S., and Zhang, H. (2020). Could environmental regulation and RandD tax incentives affect green product innovation?. Journal of Cleaner Production 258:120849. 10.1016/j.jclepro.2020.120849

[42] Song X Zhou Y Jia W. How do economic openness and RandD investment affect green economic growth?—evidence from China. Resour Conserv Recycl. (2019) 146:405–15. 10.1016/j.resconrec.2019.03.050

[43] Sun Y Mao X Liu G Yin X Zhao Y. Greener economic development via carbon taxation scheme optimization. J Clean Prod. (2020) 275:124100. 10.1016/j.jclepro.2020.124100

[44] Kirikkaleli D Adebayo TS. Do public-private partnerships in energy and renewable energy consumption matter for consumptionbased carbon dioxide emissions in India? Environ Sci Pollut Res. (2021) 28:30139–52. 10.1007/s11356-021-12692-5 33586104

[45] Khan SAR Ponce P Yu Z. Technological innovation and environmental taxes toward a carbon-free economy: An empirical study in the context of COP-21. J Environ Manage. (2021) 298:113418. 10.1016/j.jenvman.2021.113418 34426217

[46] Khan I Zakari A Ahmad M Irfan M Hou F. Linking energy transitions, energy consumption, and environmental sustainability in OECD countries. Gondwana Research. (2021). 10.1016/j.gr.2021.10.026

[47] Baniya B Giurco D Kelly S. Green growth in Nepal and Bangladesh: Empirical analysis and future prospects. Energy Policy. (2021) 149:112049. 10.1016/j.enpol.2020.112049

[48] Farhani S Shahbaz M. What role of renewable and non-renewable electricity consumption and output is needed to initially mitigate CO2 emissions in MENA region? Renew Sust Energy Rev. (2014) 40:80–90. 10.1016/j.rser.2014.07.170

[49] Bulut U. The impacts of non-renewable and renewable energy on CO2 emissions in Turkey. Environ Sci Pollut Res. (2017) 24:15416–26. 10.1007/s11356-017-9175-2 28508336

[50] Lu Y Wang S Yan C Huang Z. Robust optimal design of renewable energy system in nearly/net zero energy buildings under uncertainties. Appl energy. (2017) 187:62–71.

[51] Ahmad M Jiang P Majeed A Umar M Khan Z Muhammad S. The dynamic impact of natural resources, technological innovations and economic growth on ecological footprint: an advanced panel data estimation. Resources Policy. (2020) 69:101817. 10.1016/j.resourpol.2020.101817

[52] Fang G Wang Q Tian L. Green development of Yangtze River Delta in China under population-resources-environment-development-satisfaction perspective. Science of The Total Environment. (2020) 727:138710. 10.1016/j.scitotenv.2020.138710 32498190

[53] Sohag K Chukavina K Samargandi N. Renewable energy and total factor productivity in OECD member countries. J Clean Prod. (2021) 296:126499.

[54] Ulucak R. Renewable energy, technological innovation and the environment: A novel dynamic auto-regressive distributive lag simulation. Renew Sust Energ Rev. (2021) 150:111433.

[55] Bulut M. Effects of New Normal Life on Electricity Consumption in Covid-19 Process. JSTER. (2020), 1:4–6.

[56] Allan BB Meckling JO. Creative Learning and Policy Ideas: The Global Rise of Green Growth. Perspectives on Politics. (2021) 1–19. 10.1017/S153759272100003730886898

[57] Hultman N Sierra K Carlock G. Energy and green growth: Recasting the Options, re-envisioning sustainability. The G20'S Role In The Post-Crisis World. (2011) 35.

[58] Martinez CIP Poveda AC. The Importance of Science, Technology and Innovation in the Green Growth and Sustainable Development Goals of Colombia. Environmental and Climate Technologies. (2021) 25:29. 10.2478/rtuect-2021-0003

[59] El Sayary S Omar O. Designing a BIM energy-consumption template to calculate and achieve a net-zero-energy house. Solar Energy. (2021) 216:315–20. 10.1016/j.solener.2021.01.003

[60] Radmehr R Henneberry SR Shayanmehr S. Renewable energy consumption, CO2 emissions, and economic growth nexus: a simultaneity spatial modeling analysis of EU countries. Structural Change and Economic Dynamics. (2021) 57:13–27. 10.1016/j.strueco.2021.01.006

[61] Tao R Umar M Naseer A Razi U. The dynamic effect of eco-innovation and environmental taxes on carbon neutrality target in emerging seven (E7) economies. J Environ Manage. (2021) 299:113525. 10.1016/j.jenvman.2021.113525 34438310

[62] Chien F Sadiq M Nawaz MA Hussain MS Tran TD Le Thanh T. A step toward reducing air pollution in top Asian economies: The role of green energy, eco-innovation, and environmental taxes. J Environ Manage. (2021) 297:113420. 10.1016/j.jenvman.2021.113420 34333309

[63] Chien F Ananzeh M Mirza F Bakar A Vu HM Ngo TQ. The effects of green growth, environmental-related tax, and eco-innovation towards carbon neutrality target in the US economy. J Environ Manage. (2021) 299:113633. 10.1016/j.jenvman.2021.113633 34492439

[64] Murshed M Rahman MA Alam MS Ahmad P Dagar V. The nexus between environmental regulations, economic growth, and environmental sustainability: linking environmental patents to ecological footprint reduction in South Asia. Environ Sci Pollut Res. (2021) 28:49967–49988. 10.1007/s11356-021-13381-z 33945092

[65] Li W Elheddad M Doytch N. The impact of innovation on environmental quality: Evidence for the non-linear relationship of patents and CO2 emissions in China. J Environ Manage. (2021) 292:112781. 10.1016/j.jenvman.2021.112781 34058544

[66] Liu Y Wang A Wu Y. Environmental regulation and green innovation: Evidence from China's new environmental protection law. J Clean Prod. (2021) 297:126698. 10.1016/j.jclepro.2021.12669834510356

[67] Crippa M Solazzo E Guizzardi D Monforti-Ferrario F Tubiello FN Leip AJNF. Food systems are responsible for a third of global anthropogenic GHG emissions. Nature Food. (2021) 2:198–209. 10.1038/s43016-021-00225-9 37117443

[68] Yuping L Ramzan M Xincheng L Murshed M Awosusi AA Bah SI. Determinants of carbon emissions in Argentina: The roles of renewable energy consumption and globalization. Energy Reports. (2021) 7:4747–60. 10.1016/j.egyr.2021.07.06529756184

[69] Reisinger A Clark H Cowie AL Emmet-Booth J Gonzalez Fischer C Herrero M. How necessary and feasible are reductions of methane emissions from livestock to support stringent temperature goals? Philos Trans Royal Soc. (2021) 379:20200452. 10.1098/rsta.2020.0452 34565223 PMC8480228

[70] Swamy PA. Efficient inference in a random coefficient regression model. Econometrica. (1970) 80:311–323.

[71] Pesaran MH Yamagata T. Testing slope homogeneity in large panels. J Econom. (2008) 142:50–93.

[72] Blomquist J Westerlund J. Testing slope homogeneity in large panels with serial correlation. Econ Lett. (2013) 121:374–8.

[73] Chudik A Pesaran MH. Common correlated effects estimation of heterogeneous dynamic panel data models with weakly exogenous regressors. J Econom. (2015) 188:393–420.

[74] Bailey N Kapetanios G Pesaran MH. Exponent of cross-sectional dependence: Estimation and inference. J. Appl. Econom. (2016) 31:929–60.

[75] Bailey N Kapetanios G Pesaran MH. Exponent of cross-sectional dependence for residuals. Sankhya B. (2019) 81:46–102.

[76] Pesaran MH Schuermann T Weiner SM. Modeling regional interdependencies using a global error-correcting macroeconometric model. J Bus Econ Stat. (2004) 22:129–62.

[77] Im KS Pesaran MH Shin Y. Testing for unit roots in heterogeneous panels. J Econom. (2003) 115:53–74.

[78] Levin A Lin CF Chu CSJ. Unit root tests in panel data: asymptotic and finite-sample properties. J Econom. (2002) 108:1–24.

[79] Pesaran MH. Estimation and inference in large heterogenous panels with cross section dependence. Econometrica. (2003).

[80] Pesaran MH. A simple panel unit root test in the presence of cross? section dependence. J Appl Econom. (2007) 22:265–312.

[81] Savin I Drews S van den Bergh J. Free associations of citizens and scientists with economic and green growth: A computational-linguistics analysis. Ecological economics. (2021) 180:106878. 10.1016/j.ecolecon.2020.106878

[82] Welsby D Price J Pye S Ekins P. Unextractable fossil fuels in a 1.5 C world. Nature. (2021) 597:230–4. 10.1038/s41586-021-03821-8 34497394

[83] Agbulut Ü Saridemir S. A general view to converting fossil fuels to cleaner energy source by adding nanoparticles. Int J Ambient Energy. (2021) 42:1569–74. 10.1080/01430750.2018.1563822

[84] Urbaniec M Tomala J Martinez S. Measurements and Trends in Technological Eco-Innovation: Evidence from Environment-Related Patents. Resources. (2021) 10:68. 10.3390/resources10070068

[85] Pesaran MH. Estimation and inference in large heterogeneous panels with a multifactor error structure. Econometrica. (2006) 74:967–1012.

[86] Eberhardt M Teal F. Productivity Analysis in Global Manufacturing Production. DEGIT Conference Papers c015_019, DEGIT, Dynamics, Economic Growth, and International Trade. (2010)

[87] Kapetanios G Pesaran MH Yamagata T. Panels with non-stationary multifactor error structures. J Econom. (2011) 160:326–48.

[88] Cavalcanti TVDV Mohaddes K Raissi M. Growth, development and natural resources: New evidence using a heterogeneous panel analysis. Q Rev Econ Finance. (2011) 51:305–18.

[89] Granger CW. Investigating causal relations by econometric models and cross-spectral methods. Econometrica. (1969) 80:424–38.

[90] Dumitrescu EI Hurlin C. Testing for Granger non-causality in heterogeneous panels. Econ Modell. (2012) 29:1450–60.

